# Motor system recruitment during action observation: No correlation between mu-rhythm desynchronization and corticospinal excitability

**DOI:** 10.1371/journal.pone.0207476

**Published:** 2018-11-15

**Authors:** Olivia M. Lapenta, Elisabetta Ferrari, Paulo S. Boggio, Luciano Fadiga, Alessandro D’Ausilio

**Affiliations:** 1 The MARCS Institute for Brain, Behaviour & Development, Western Sydney University, Penrith NSW, Australia; 2 Social and Cognitive Neuroscience Laboratory, Center for Health and Biological Sciences, Universidade Presbiteriana Mackenzie, São Paulo SP, Brazil; 3 Department of Languages and Literatures, Communication, Education and Society, University of Udine, Udine, Italy; 4 Section of Human Physiology, Università di Ferrara, Ferrara, Italy; 5 Center for Translational Neurophysiology of Speech and Communication, Fondazione Istituto Italiano di Tecnologia, Ferrara, Italy; University of California Los Angeles, UNITED STATES

## Abstract

Observing others’ actions desynchronizes electroencephalographic (EEG) rhythms and modulates corticospinal excitability as assessed by transcranial magnetic stimulation (TMS). However, it remains unclear if these measures reflect similar neurofunctional mechanisms at the individual level. In the present study, a within-subject experiment was designed to assess these two neurophysiological indexes and to quantify their mutual correlation. Participants observed reach-to-grasp actions directed towards a small (precision grip) or a large object (power grip). We focused on two specific time points for both EEG and TMS. The first time point (t1) coincided with the maximum hand aperture, i.e. the moment at which a significant modulation of corticospinal excitability is expected. The second (t2), coincided with the EEG resynchronization occurring at the end of the action, i.e. the moment at which a hypothetic minimum for action observation effect is expected. Results showed a Mu rhythm bilateral desynchronization at t1 with differential resynchronization at t2 in the two hemispheres. Beta rhythm was more desynchronized in the left hemisphere at both time points. These EEG differences, however, were not influenced by grip type. Conversely, motor potentials evoked by TMS in an intrinsic hand muscle revealed an interaction effect of grip and time. No significant correlations between Mu/Beta rhythms and motor evoked potentials were found. These findings are discussed considering the spatial and temporal resolution of the two investigated techniques and argue over two alternative explanations: i. each technique provides different measures of the same process or ii. they describe complementary features of the action observation network in humans.

## Introduction

Action understanding is a fundamental skill [1] for which sensorimotor brain activities may provide critical support (for a review see [2]). During action observation the activation of sensory and motor circuits necessary to execute the same action may provide an anticipatory mechanism to constrain sensory processing [3].

Human neurophysiological research in this area has benefited from the use of two complementary techniques, transcranial magnetic stimulation (TMS) and electroencephalography (EEG). The amplitude of motor evoked potentials (MEPs), elicited by single pulse TMS over the primary motor cortex, provides a direct index of corticospinal recruitment. During action observation MEPs are modulated in accordance to the expected changes during action execution [4] (for a review please see [5, 6, 7]). In parallel, EEG research has shown that event-related synchronization (ERS) and desynchronization (ERD) of the sensorimotor alpha band, named as Mu rhythm, is associated with action performance, imagery and observation [8, 9, 10] (for a review see [11]). Therefore, modulation of both the central Mu ERD [12, 13] and of corticospinal excitability [14, 15] during action observation may reflect activity of a mirror-like system present in humans [16, 17].

Although both measures have been well documented during action observation and execution, only one study attempted to establish a correlation between the two of them [15]. Lepage and colleagues (2008) found no correlation between MEPs amplitude and Mu power recorded during rest, observation, execution and imagery of a ball squeezing action. Instead, they found a correlation between MEPs and Beta rhythm during rest and action execution conditions. Lepage and colleagues analyzed the EEG rhythms as a steady oscillatory pattern during two cycles of the squeezing action, within a 2 seconds window. Furthermore, TMS was delivered randomly within a one second window [15]. These experimental features could have introduced some uncertainty in the measurement of both indexes. In fact, the TMS study did not exploit the accurate temporal resolution granted by this technique. At the same time, the EEG analyses did not consider that neural oscillatory effects could be modulated for the observation of different phases of an ongoing action. Indeed, Mu and Beta power might be modulated differently across different phases of an ongoing action. Specifically, desynchronization starts very soon, peaks during movement to later rebound after the end of the action (i.e., ERS) [18]. At the same time, corticospinal excitability is differentially modulated during the different phases of hand opening and closing observation [14, 19], while it is also influenced by the ongoing alpha and beta power [20, 21]. As a consequence, to fully evaluate the relationship between Mu/Beta ERD and corticospinal excitability, the two measures need to be time-locked to specific kinematic landmarks of the observed actions.

Herein we designed a classical reach-to-grasp action observation task directed towards a small and a large object, thus requiring a precision or a power grip, respectively. We collected EEG and MEPs from the First Dorsal Interosseous muscle, in the same subjects in two separate sessions. Our main goal was to evaluate corticospinal excitability and Mu or Beta rhythms modulation at different critical time points during the action and then correlate these measures. The first time point was aligned with maximum hand aperture (t1), when maximal corticospinal modulation has been previously reported [14]. The second time point was aligned to the end of the action where we observed in our data, at the group level, that the Mu rhythm resynchronizes (t2). Transitive actions (object-directed) were chosen due to previous studies suggesting greater modulatory effects on both Mu rhythm [18, 22] and corticospinal excitability [23]. Our prediction is that the two measures bear no relationship with each other mostly due the inherent temporal and spatial sensitivity differences between them.

## Methods

### Participants

Eighteen right-handed subjects (11 female; mean age = 27.22, SD = 4.32, age range: 22–39 years old) were included in the study after verifying compliance with inclusion criteria for the safe use of TMS [24]. They had normal or corrected-to-normal visual acuity, and were naive to the purposes of the study. The study was conducted according to the standards of the Declaration of Helsinki (World Medical Organization, 1996) and was approved by the local ethics committee (protocol 32/08 del 2/10/2008 –Azienda Sanitaria Locale (ASL) 3 Genova). Participants gave their written informed consent prior to performing the experiment and were remunerated for their participation.

Estimation for sample size was run with G*Power (V. 3.1.9.3), based on data from the 12 subjects collected by Lepage and colleagues [15]. The non-significant correlation between MEPs size and mu power (8-13Hz), during action observation (-0.186) was set as ρH0 and the correlation value observed in the action execution condition (exploratory analyses; -0.75) was set as ρH1. The power analyses showed that to demonstrate a significant correlation between TMS and EEG indexes of action observation effects, which are as evident as those shown during action execution in Lepage and colleagues [15], the needed sample size is of at least 15 participants (two tails; alpha = 0.05; beta = 0.2).

### Stimuli

We recorded goal-directed action video stimuli performed with the right hand, lasting approximately 2100ms, divided in two categories: precision grip and power grip. Both videos started with the presentation for 600ms of a still image depicting the lateral view of an upper limb resting on one side of a black table and two white spheres (2 cm and 10 cm in diameter, the to-be-grasped objects) positioned on the opposite side of the table. After the first 600ms, participants were presented with a reaching-grasping-lifting action directed towards one of the two objects. Thus, the two videos differed according to the goal of the action (precision grip or power grip). In order to introduce some variability across the stimuli we used four different actors (2 males and 2 females). During video recording, actors’ kinematics was acquired by a 9-cameras *Vicon* system (Vicon Motion Systems, Oxford, UK). Kinematic recordings were done at 100 Hz, with markers attached to the ulnar styloid, pinkie knuckle, index knuckle, thumb nail, index finger nail, 3^rd^ finger nail, 4^th^ finger nail, 5^th^ finger nail, 5^th^ finger knuckle). Kinematic data was analyzed using Matlab (The MathWorks Inc., Natick, MA, USA). We selected a total of 8 videos, four of precision grips and four of power grips, in which the peak of transport velocity and the maximum hand aperture overlapped between actors as much as possible. Maximum hand aperture occurred approximately 1500ms after video onset.

### TMS

TMS stimulation was delivered through a figure-of-eight coil (70 mm) connected to a Magstim 200 stimulator (Magstim Co., Whitland, UK). EMG was recorded from the right First Dorsal Interosseous (FDI), using a standard tendon-belly montage with Ag/AgCl electrodes. Data was amplified via wireless electromyography (ZeroWire EMG, Aurion), band-pass filtered (50–1000 Hz), and digitized at 2000 Hz by an analog-to-digital acquisition board (CED Power1401, Cambridge Electronics, UK; Signal software version 4). A pre-stimulus recording of 200 ms was used to check for the presence of EMG activity before TMS pulse. TMS coil positioning over the left primary motor cortex was optimized to induce the largest responses at the lowest intensity, with the handle pointing backwards, 45° from the midline. Coil position was kept constant through a coil holder, whereas the head was stabilized using a 3-points arc-shaped holder. Resting Motor Threshold (rMT) was defined as the lowest stimulation intensity capable of evoking MEPs of at least 50μV, 5 times out of 10 (24). Participants’ right hand was kept on a pillow in relaxed prone position. During the experiment, single pulse TMS was applied over the identified hotspot, with an intensity of 120% of the rMT. TMS was triggered by the PC parallel port controlled by E-prime 2.0 software (E-prime, http://www.pstnet.com/eprime.cfm).

### EEG

The EEG data was recorded by means of a 32 Ag/AgCl active electrodes cap (ActiCap, Brain products, München, Germany) placed over the scalp according to the international 10–20 system. Data was amplified using Brain Amp MR plus (Brain Products, München, Germany) and acquired via the Brain Vision Recorder software (Brain Products, München, Germany) at a 500Hz sampling rate, in continuous mode. EEG signal markers for the onset of video were sent through the parallel port controlled by E-prime 2.0 (E-prime, http://www.pstnet.com/eprime.cfm). Electrode impedance was kept below 5KΩ throughout the experiment. Two electrodes, placed at the outer canthi of each eye, recorded horizontal eye movements (EOG). All data was stored for offline analyses.

### Procedure

Upon arrival, all participants were informed about the procedure and provided written informed consent. The two tasks were performed according to a fixed order (EEG first and TMS after one month). The EEG protocol was conducted before TMS as we intended to use the EEG results to guide the choice of the TMS pulse timing (t2). All participants completed both experiments. After the initial preparation (EEG cap montage or TMS hot-spot localization and motor threshold assessment), participants started the appropriate task. They sat on a comfortable chair at a distance approximately of 58 cm from a 17” LCD computer monitor (1024 × 768 pixels; refresh rate: 60 Hz). Both tasks were controlled using E-prime scripts.

#### EEG experiment

The task comprised 64 trials (32 trials of precision grip and 32 trials of power grip). Trials were presented divided in 4 experimental blocks, to check electrodes impedance between blocks. Each trial started with a central black fixation cross over a grey background (500ms) followed by the video presentation. As soon as the video ended, a black screen was presented for 2000ms. This interval was defined considering the Mu and Beta rhythms rebound effect (i.e., resynchronization) that takes at least 1000ms to return to baseline. In addition, in 12 occasions, randomly included among trials, subjects had to report if the last observed video was the repetition of the previous one. Participant had to respond as soon and as accurately as possible within 1000 ms, by pressing a key with their left hand. Whenever an attentional question was presented it was also followed by additional 2000ms of black screen before the next video presentation. EEG was recorded during the whole experiment. The total duration of the procedure, including preparation, was approximately 30min.

#### TMS experiment

**Fig 1:**
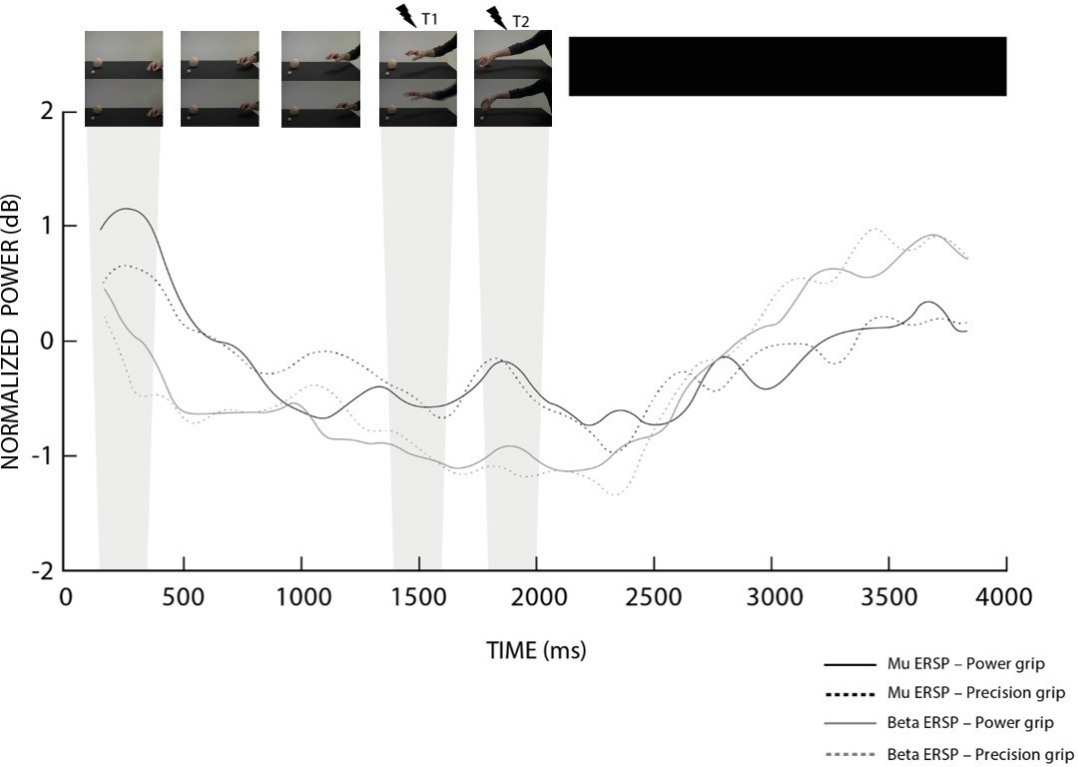
Time course of Mu and beta EEG rhythms. The graph shows the spectral perturbation of Mu (8–12 Hz) and beta (15–23 Hz) bands recorded in the left hemisphere (C3). The ‘X’ axis represent the time in milliseconds; whereas T1 (1500ms) and T2 (1900ms), represent the instants of TMS pulses and grey rectangles highlight the correspondent time-bin of EEG analyses. The ‘Y’ axis represents the log transformed ratio of the power at each time point relative to the average power of the respective condition. At the top of the figure we illustrate in a few frames the action presented in the video clips.

The task comprised 40 experimental trials and 40 catch trials (without TMS), for a total of 80 trials. Catch trials were included to control the effects of adaptation and to prevent subjects’ prediction of TMS timing. Trials were intermingled by 5 checks of the coil position. In experimental trials, TMS was delivered randomly in one of the two pre-established time points; specifically, 1500ms (t1) and 1900ms (t2) after action onset. The first time point (t1) corresponded to maximum hand aperture/reaching velocity, whereas t2 corresponded to the end of object lifting. In order to determine t2, we visually inspected the modulation of the Mu band, at the group level, acquired in the EEG experiment, looking for a time-window of synchronization during the presentation of the two action video types (please see Fig 1). Each trial started with a central black fixation cross over a grey background (500ms) followed by the video presentation. After the end of the video, a black screen was presented for 3000ms. Twelve attentional questions were randomly included among trials (see above). Due to TMS safety issues and to resume basal corticospinal excitability a minimum of 10s intervals between TMS pulses was established. The total duration of the TMS procedure, including preparation, was approximately 35 min.

### Analyses

#### EEG analyses

EEG data was pre-processed using the EEGLab toolbox (http://sccn.ucsd.edu/eeglab/) in the MatLab software environment. Data pre-processing was performed as follows: a) 0.1 Hz High-pass, 100 Hz Low-pass and a Notch filter; b) artefact removal through visual inspection by Independent Component Analysis (ICA) considering time, topographic and spectral distribution of the components; c) re-reference of scalp potentials using the average of all connected electrodes; d) data segmentation in epochs of -400ms before and 4400 ms after the beginning of each video.

After pre-processing, the spectral representations of precision and power grasp trials were computed. For this purpose, a Morlet transformation was applied to each trial using 7 cycles to analyze oscillatory activity in the range of 1–30 Hz using 1 Hz step [25]. The spectral representation of the data in the electrodes of interest (i.e., C3) was used to select the most reactive frequency range of Mu and Beta bands in our sample (Fig A in S1 File). The most reactive frequency range for the Mu band was determined as 8–12 Hz and for the Beta band as 15–23 Hz. Next, we plotted the Mu power throughout the trial window to verify peaks of ERD and/or ERS and guide the choice of timing (t2).

For the EEG analyses two time-bins were considered. The first one (t1) from 1400 to 1600ms, represent a 200ms window centered on maximum hand aperture and arm peak velocity at 1500ms. The second one (t2) from 1800 to 2000ms, corresponding to an ERS peak representing the moment when the grasping action was concluded and the object was lifted (Fig 1). Importantly, 200ms bins is a duration previously used and shown sensitive to oscillations of alpha and beta activity [18].

The power change, defined as Event-Related Spectral Perturbation (ERSP) in the time-bins of interest, was computed based on the logarithmic ratio of the power within bins of interest (t1 or t2) with respect to the power of the whole trial (as a baseline) of the respective condition (precision or power grip). We extracted the resulting values of t1 and t2 for power and precision grip in C3 and C4 electrodes to compute statistical analyses. We performed 2 separate 2x2x2 repeated measures ANOVAs, with Mu and Beta power as dependent variables. The factors were Time (t1 *vs* t2), Condition (precision *vs* power) and Electrode (C3 *vs* C4). Newman–Keuls corrected post-hoc tests were conducted to further explore significant data.

#### TMS analyses

First, we eliminated trials containing EMG activity before TMS (time window -200 to 0ms) and outlier data points above and below 2SD from the mean (on average we eliminated 3.5, SD = 3 trials per subject, 8.7%). MEPs were z-score transformed at the single subject level, then we averaged values for each time point and condition. We then performed a 2x2 repeated measures ANOVA with MEPS amplitude as dependent variable and factors Time (t1, t2) and Condition (precision, power). Newman–Keuls corrected post-hoc tests (p<0.05) were used to further explore significant factors and interactions.

#### Correlational analyses

Lastly, we performed Pearson Correlation analyses between the normalized MEPs value and Mu or Beta ERSP during each condition (precision, power) and times of interest (t1, t2). Because TMS was delivered to the left hemisphere, Mu and Beta power recorded from the electrode C3 were used for the correlation analyses.

Finally, considering that ongoing EEG oscillatory dynamics may modulate upcoming MEPs size [20; 21], we ran a further correlation analyses by considering additional time-windows preceding and following the TMS at t1 (1300-1500ms and 1500-1700ms) and t2 (1700-1900ms and 1900-2100ms). This analysis was employed to verify whether effects evidenced by the two techniques suffered from a relative temporal lag.

## Results

### EEG

**Fig 2:**
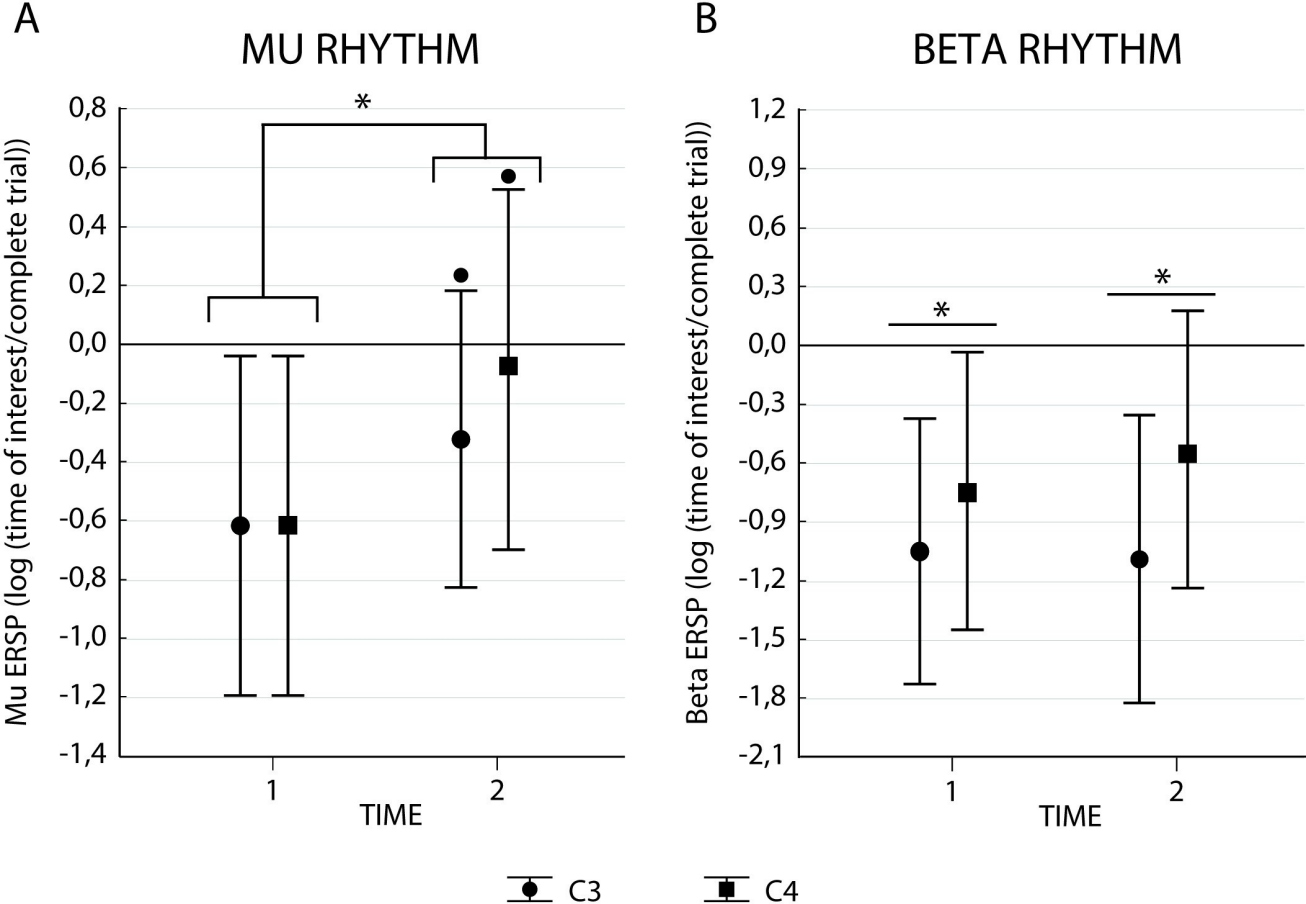
Mu and beta ERSP results. Panel A represents data for Mu, while Panel B shows the Beta ERSP. Bars indicate confidence interval (95%), (*) indicate significant differences with p<0.05 and (•) a trend p<0.1.

The ANOVA on Mu rhythm revealed a main effect of Time (F_1,17_ = 8.75, p < 0.01, *η*_*p*_^*2*^ = .34) and no significant main effect of Electrode (F_1,17_ = 1.15, p = 0.31, *η*_*p*_^*2*^ = .06) or Condition (F_1,17_ = 0.28, p = 0.6, *η*_*p*_^*2*^ = 0.02). Concerning interactions, there was only a trend for the interaction Electrode*Time (F_1,17_ = 3.69, p = 0.07, *η*_*p*_^*2*^ = .18). Mean values and standard error for Mu and Beta rhythms are provided in Table 1. As can be seen in Fig 2A, we found an ERD during the first time-bin, followed by a relative resynchronization at the second time-bin. Qualitatively speaking, the non-significant interaction trend suggests that the synchronization at t2 was more prominent in C4 electrode.

**Table 1:** Means and standard error of mean (SE) of Mu and beta ERSP for each condition (precision grip *vs* power grip) and time bin (t1 *vs* t2).

		C3				C4			
		mu		Beta		mu		beta	
		mean	SE	Mean	SE	mean	SE	mean	SE
**Precision**	**t1**	-0.610	0.193	-1.031	0.242	-0.672	0.246	-0.808	0.260
	**t2**	-0.351	0.196	-1.219	0.302	-0.123	0.201	-0.661	0.264
									
**Power**	**t1**	-0.622	0.241	-1.075	0.249	-0.556	0.206	-0.687	0.250
	**t2**	-0.295	0.206	-0.964	0.211	-0.051	0.248	-0.417	0.226

The ANOVA on Beta rhythm revealed a main effect of Electrode (F_1,17_ = 12.79, p = 0.002, *η*_*p*_^*2*^ = 0.43), no significant main effect of Time (F_1,17_ = 0.45, p = 0.51, *η*_*p*_^*2*^ = 0.03), or Condition (F_1,17_ = 1.98, p = 0.18, *η*_*p*_^*2*^ = 0.10) nor interaction effects. As can be seen in Fig 2B, irrespective of time, there was a higher Beta band desynchronization in C3 when compared to C4.

In acknowledgement of the difficulty to choose an ideal baseline [26], we have also performed EEG analysis using the 200ms of static hand presentation as baseline. Specifically, the time window from 150–350 ms of the video. This analysis, for Beta rhythm, resulted in no significant main effects and no interaction effects. For the Mu rhythm, analysis using static hand as baseline resulted in the same effects as using whole trial as baseline. Namely, main effect of time (p = 0.009) and a trend for the interaction electrode*time (p = 0.071). As baseline conditions have been argued to arise potential confound [26] hereafter we will refer only to the data normalized using the whole trial as baseline. In principle, this choice is more conservative as it is supposed to let emerge only transient and large power modulations.

### TMS results

**Fig 3:**
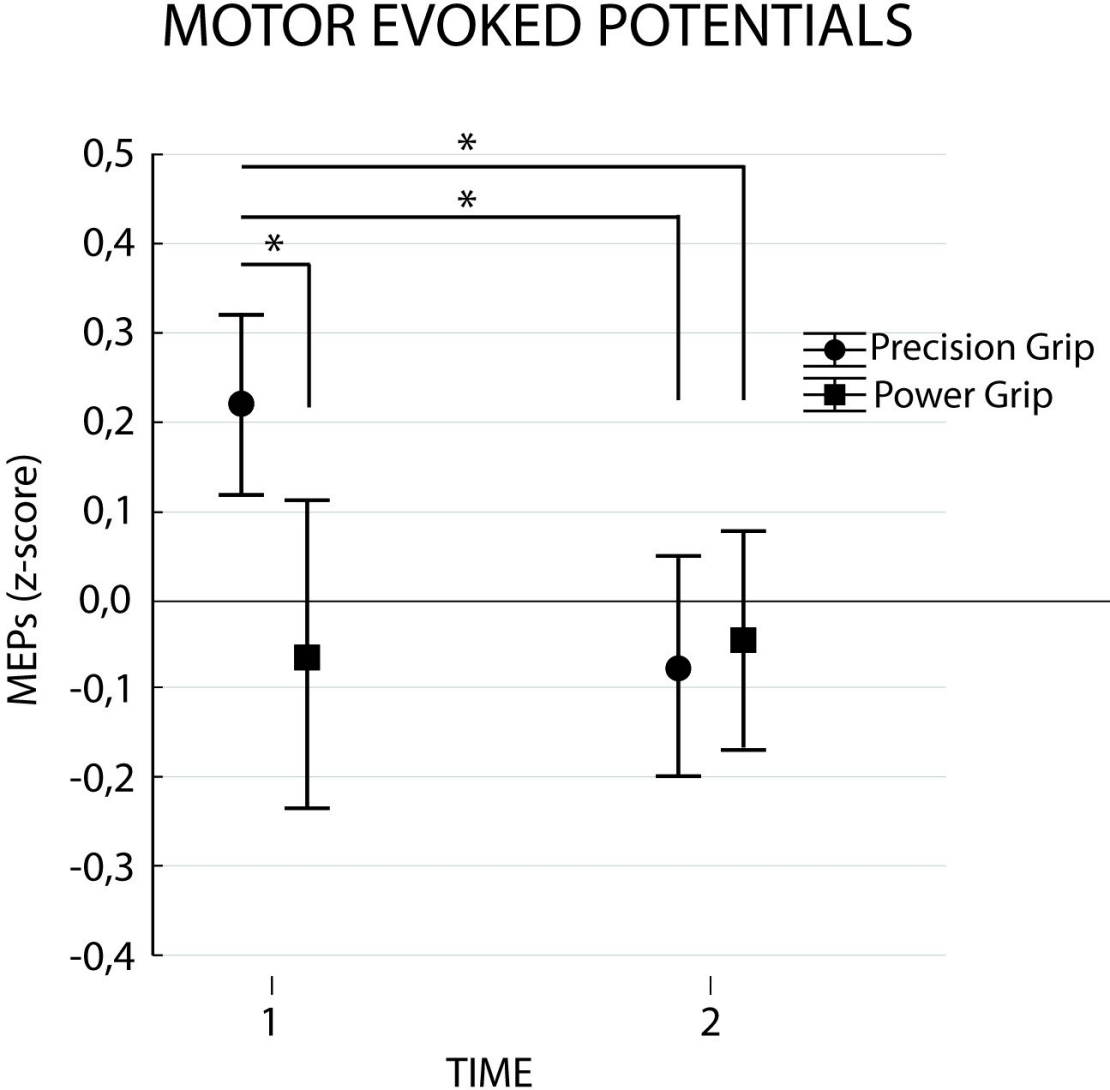
Corticospinal excitability results. Normalized MEPs amplitudes for each condition (Precision vs Power grip) in time points 1 (maximum hand aperture) and 2 (movement end). Bars represent confidence interval (95%). Asterisks indicate significant comparisons (p<0.05).

The ANOVA performed on MEPs revealed a significant interaction Condition*Time (F_1,17_ = 4.77, p = 0.04 *η*_*p*_^*2*^ = 0.22). The factors Condition (F_1,17_ = 2.63, p = 0.12, *η*_*p*_^*2*^ = 0.13) and Time (F_1,17_ = 2.56, p = 0.13, *η*_*p*_^*2*^ = 0.13) did not show any significance. As shown in Fig 3 and confirmed by post-hoc analysis, observation of precision grip yielded larger MEPs at t1 when compared to t2 (p = 0.04) and also when compared to power grip at t1 (p = 0.03) and t2 (p = 0.01) (see Table 2).

**Table 2:** Means and standard errors of normalized MEPs amplitude for each condition (precision grip *vs* power grip) and time (t1 *vs* t2).

		Mean	SE
**Precision**	**t1**	0.215	0.05
	**t2**	-0.076	0.06
**Power**	**t1**	-0.066	0.09
	**t2**	-0.054	0.06

### EEG-TMS correlation analyses

First analysis showed no correlations between Mu or Beta rhythms and MEPs amplitude. Finally, correlations between the two additional time-windows preceding and following the TMS pulses also showed no significant effects (see Table 3).

**Table 3:** Pearson correlation for corrected Mu or beta rhythm and MEPs. Table show r and p values for Precision and Whole grasp conditions in times 1 and 2 (Fig B in S1 File) and also for the time-windows preceding and following the TMS at t1 (1300-1500ms and 1500-1700ms) and at t2 (1700-1900ms and 1900-2100ms).

			r	p
Mu Rhythm	Precision	t1	0.291	0.227
		t2	-0.119	0.627
		1300-1500ms	0.907	0.720
		1500-1700ms	0.033	0.897
		1700-1900ms	-0.007	.977
		1900–2100 ms	-0.065	0.799
	Power	t1	-0.103	0.674
		t2	0.03	0.904
		1300-1500ms	-0.334	0.176
		1500-1700ms	-0.117	0.643
		1700-1900ms	-0.058	0.820
		1900–2100 ms	0.134	0.595
Beta Rhythm	Precision	t1	0.148	0.557
		t2	-0.068	0.789
		1300-1500ms	0.051	0.840
		1500-1700ms	0.137	0.586
		1700-1900ms	-0.096	0.705
		1900–2100 ms	-0.247	0.324
	Power	t1	0.124	0.657
		t2	-0.114	0.653
		1300-1500ms	0.074	0.772
		1500-1700ms	0.235	0.355
		1700-1900ms	-0.356	0.147
		1900–2100 ms	0.398	0.102

## Discussion

Action observation has shown to activate the same neural network involved in action execution. This evidence has been derived from neurophysiological techniques allowing very different spatial and/or temporal specificity.

### EEG

In agreement with Coll and coworkers [27], we showed that the Mu rhythm was not modulated by the observed grasp-type. Considering that power modulations of EEG rhythmic activities inform about the global activity of huge neural population, previous studies showed dissociable activities only for different effectors (i.e. leg *vs*. arm [27, 28, 29]). However, in line with Avanzini et al. [18] we found that Mu modulation depends on the phase of the observed action, i.e. in our experiment there was a greater ERD during observation of maximum hand aperture.

Differently, the Beta rhythm showed no significant differences between time and grasp-type. This is in line with the idea that each frequency band likely reflects different aspects of action observation and execution processing [16]. Specifically, Beta band modulations **is** related to motor functions such as sustained muscle contraction or voluntary movement, being thus linked to motor control of the ongoing action [30]. In contrast, modulations of the Mu band reflect somatosensory processes associated with action observation [27] and motor learning through observation [31].

In our study, Mu rhythm rebounds bilaterally at the end of the movement with a trend for a higher ipsilateral re-synchronization (at C4). It has been proposed that contralateral (e.g., [32, 33, 34]) or bilateral (e.g., [35, 36]) rhythmic modulations vary according to the experimental task [37]. A previous study testing neuromodulatory effects over the Mu rhythm during right hand action observation demonstrated that anodal stimulation over C3 led to synchronization and desynchronization measured at C3 and C4, respectively, such findings are discussed considering that the ipsilateral hemisphere is recruited when the contralateral motor area cannot process the information [32]. Accordingly, additional recruitment of the ipsilateral hemisphere in unimanual tasks has been related to task complexity during motor execution [38]. However, such studies did not compare inter-hemispheric Mu oscillations over the time course of an observed action.

Altogether, our findings suggest that initial action phases may recruit bilateral motor areas to provide greater effectiveness in extracting movement features. We speculate that after the action has been decoded, the additional ipsilateral hemisphere activation might no longer be necessary, thus leading to a greater rebound. In any case, our task consisted in monitoring the unfolding of a relatively simple action and indeed results are aligned with previous findings accounting task complexity as a major factor in determining the degree of inter-hemispheric cooperation [39]. Regarding the Beta rhythm, ERD effects were more prominent over the left hemisphere. Similar hemispheric differences have been recently demonstrated in an elegant study where participants watched tennis movements and were requested to predict the tennis ball direction [33]. This lateralized desynchronization pattern was associated with a higher accuracy in the behavioral task, suggesting a correlation with goal prediction. The lack of time-dependent Beta modulation in our study reinforces previous interpretations about the independence of Mu and Beta rhythms (for a review see [40]).

### TMS

As expected, corticospinal excitability during action observation differentiates between precision and power grips when tested in correspondence of the maximum hand aperture; showing significantly larger FDI muscle MEPs during precision grip. Different motor neural populations are recruited depending on grip type, and such differences can be evidenced by MEPs size [41, 7]. Precision grip requires more accuracy in finger positioning and consequently greater control on muscle activity during execution [42]. Moreover, as shown by cortical stimulation and recording experiments performed on monkeys [43, 44] and humans [45], precision grip has a larger cortical representation than power grip. Concerning the action phase, results match previous reports [14, 41]. Larger corticospinal modulations during mid-phases of the action indicate that one important function of this system could be to predict action outcomes [46]. In general, our results are in line with previous studies suggesting that corticospinal excitability modulation can probe the activity of the action observation network with high temporal (i.e. movement phase) and spatial (i.e. grip type configuration) accuracy [5, 6].

### Correlation between EEG and TMS data

When comparing TMS and EEG results, we found no correlation between MEPs amplitudes with Mu or Beta power. This result agrees with a previous report [15] although with important differences. While in the above mentioned study the observed action consisted in the cyclic squeezing of a ball, here we implemented a reaching-grasping action towards objects of different sizes, both present on a table. Therefore, the goal is not specified since the beginning, but it gradually emerges while the action unfolds. This fact potentially engages the mirror system and its possible role in prediction [18, 46]. More importantly, we also anchored the time windows of exploration to two critical functional landmarks. The first was based on the actors’ motion kinematics corresponding to maximal action observation effects [14, 41]. The second was aligned to the synchronization of EEG activity after the first time point. Therefore, our design allowed us to correlate the effects observed at two functionally different time points, across techniques. This analysis showed no significant correlations between the two measures. No correlation was observed even when we considered EEG windows preceding and following t1 and t2.

In general, the lack of a clear and statistically reliable correlation pattern suggests that EEG and TMS measures of action observation effects have at least a foggy relationship at the single subject level. Therefore, the question is whether the lack of consistent correlations is due to the inherent differences in resolution of the two techniques or because they target different aspects of the same mechanism which are at best loosely related.

The different spatio-temporal resolution of each method may be the first obvious reason for the lack of correlation. TMS measures temporally resolved muscle-level corticospinal excitability while ERSPs report cortical network-wide slow oscillations with smaller temporal and spatial resolution. Furthermore, there is also a spinal contribution to the modulation of MEPs amplitudes [47], which is fundamentally reduced in EEG recordings. A recent meta-analysis reported Mu rhythm suppression over central areas during overt action execution, while action observation modulates Mu oscillations also at more frontal and parietal areas [17]. This may suggest that during action observation Mu modulation reflects system–wide slow cortical modulations that may not correlate with the temporally fine-grained description allowed by corticospinal excitability modulations.

Importantly, some limitations of our study should be accounted. Firstly, participants undertook the experiments in fixed order, first EEG and then TMS. As expertise [48] and learning of a novel action [49] have been demonstrated to respectively modulate MEPs and EEG rhythmic activity, it is arguable that a fixed experimental order could introduce a potential confound. Although the present actions are very familiar (reach to grasp an object) and the two sessions were separated by one month interval, we acknowledge that it is not possible to completely exclude a learning effect. A second limitation is that we did not select individual Mu rhythm range (e.g., [31]). Instead we selected the Mu range at group level, which also coincided with the most usual selected range of 8–12 Hz (e.g., [9, 32, 33]). Although results are largely aligned with the literature, we acknowledge that this could potentially increase a type II error.

## Conclusions

The neural processes triggered by action observation have been proposed to be layered in several functionally interconnected levels. These levels are (i) the muscular level–decodes the pattern of muscle activity necessary to perform the action; (ii) the kinematic level–maps the effector movement in time and space; (iii) the aim level—includes transitive or intransitive short-term goal; (iv) the intention level–regards the long-term purpose of the action [50, 51, 52]. Future studies may indeed combine these techniques to obtain complementary information and thus allow the investigation of the hierarchical organization of action observation-induced effects.

Our results are to be read as a cautionary note towards the overly simplistic generalization of results across techniques granting very different potential. In fact, it is still to be understood if differences in temporal and spatial resolution explains the lack of correlation, or the two techniques could still be providing different measures of the same neural process. Given the obvious differences in temporo-spatial resolution granted by the two techniques, we believe the first hypothesis to be the most likely.

## Supporting information

S1 File **Fig A.** Time-frequency power spectrum plot. Illustrates, at group level, the power spectrum for Precision (P) and Power (W) conditions at C3 electrode. **Fig B.** Correlation between Mu and Beta rhythms and MEPs. B1. Precision condition at t1; B2. Precision condition at t2; B3. Power condition at t1; B4. Power condition at t2.
(PDF)
