# Supplementary figures and images for: Motor system recruitment during action observation: No correlation between mu-rhythm desynchronization and corticospinal excitability
Source: PLoS One. 2018 Nov 15;13(11):e0207476. doi: 10.1371/journal.pone.0207476 (PMC6237396; doi:10.1371/journal.pone.0207476)

## Supplementary Material

### 1. Fig A

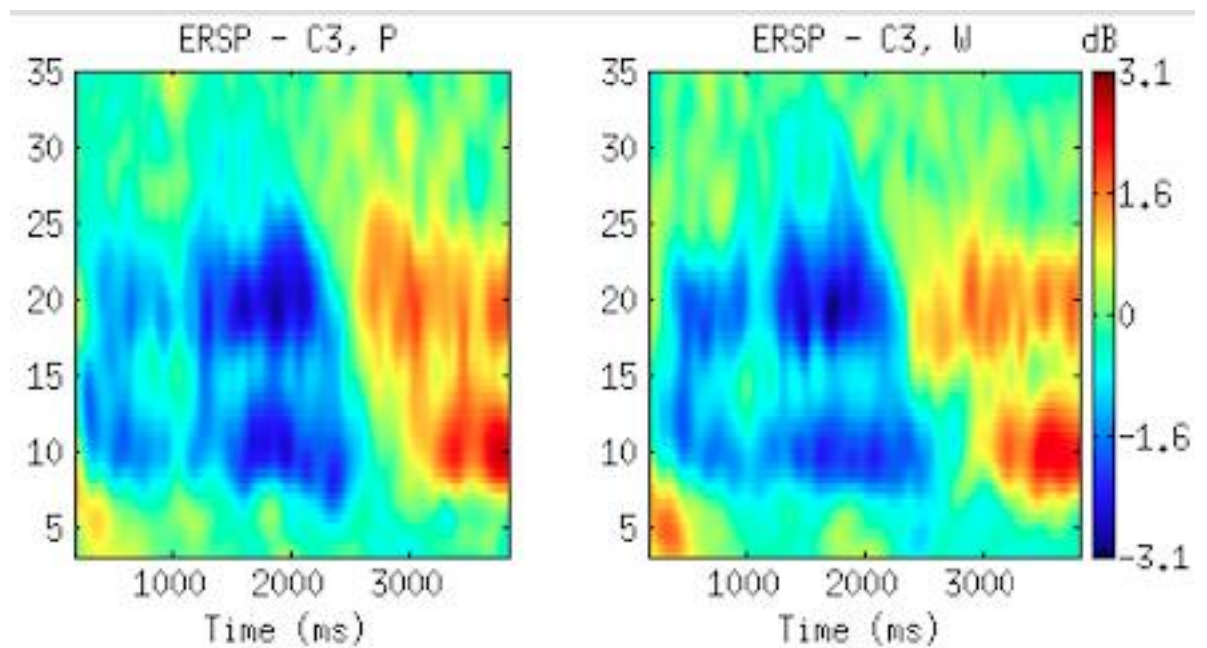

### 2. Fig B

#### B1

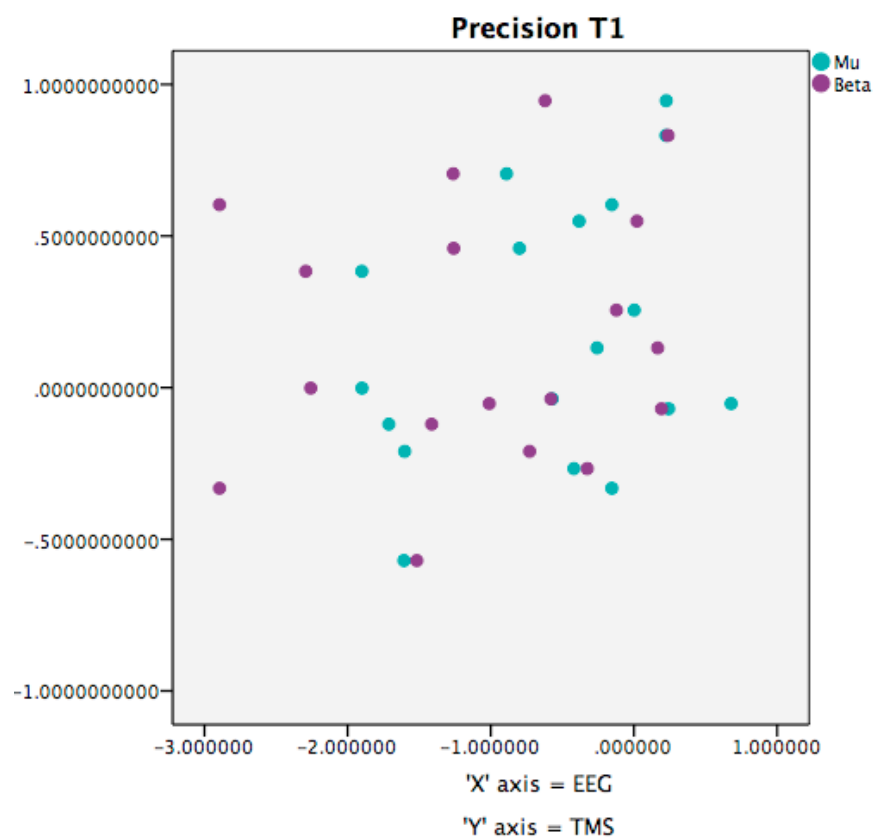

B2

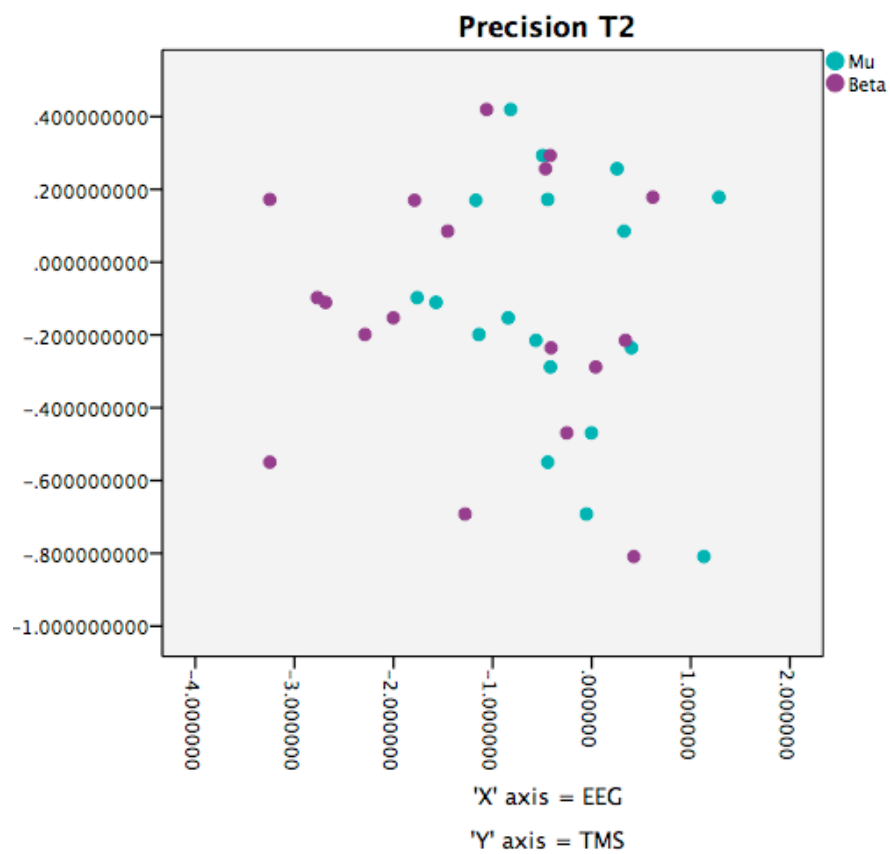

B3

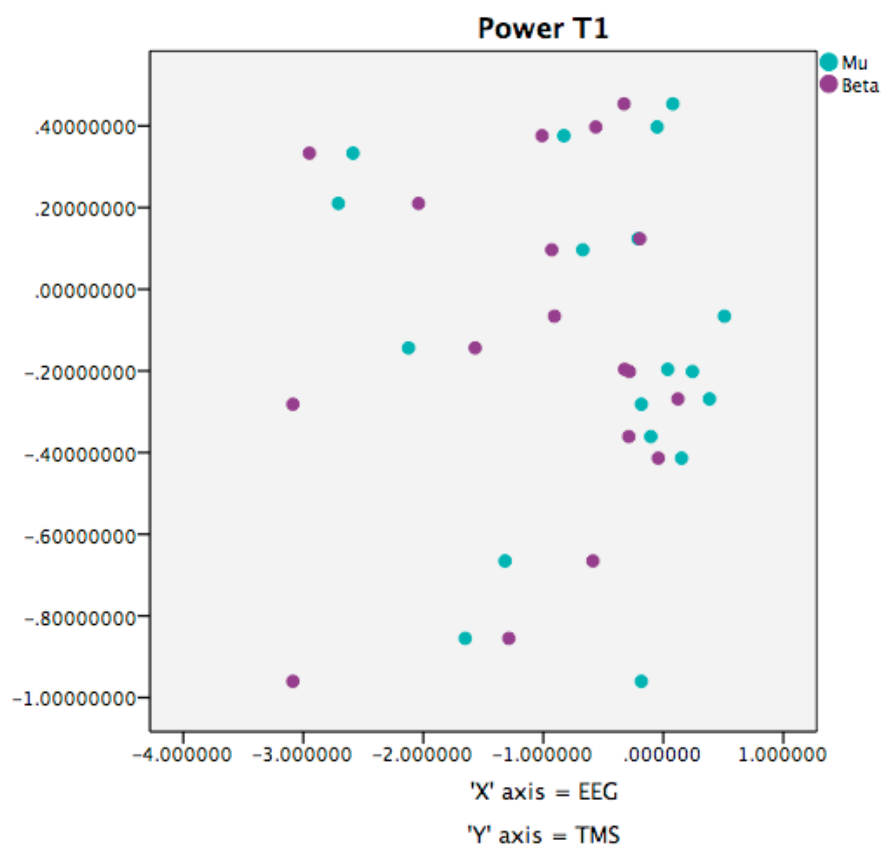

B4

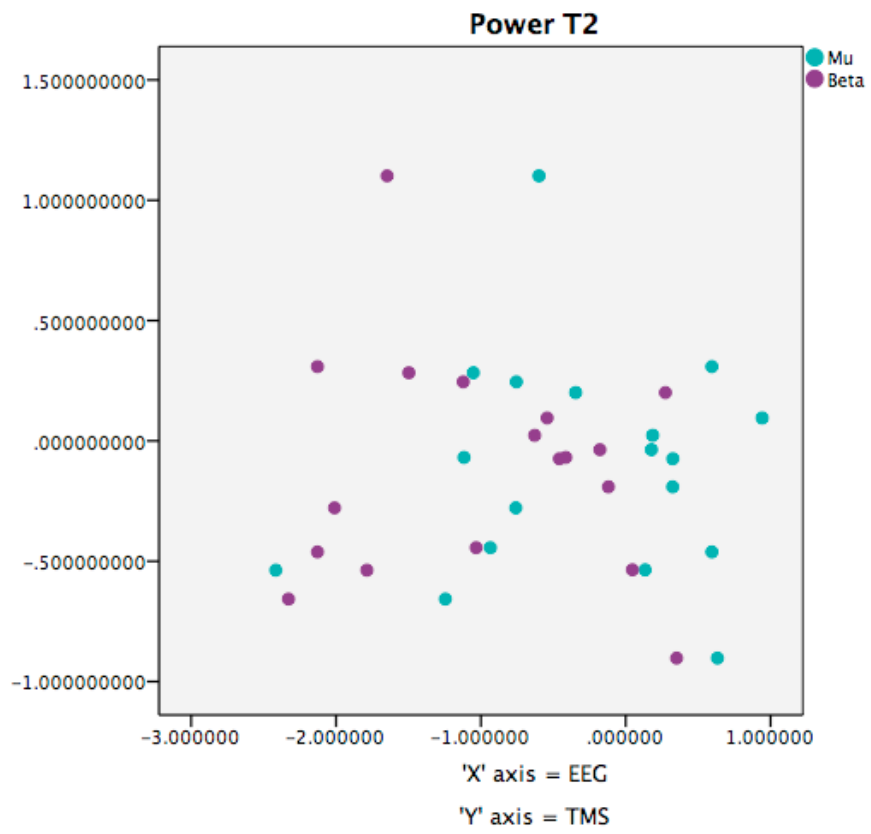

Supplement: S1 File — Fig A. Time-frequency power spectrum plot. Illustrates, at group level, the power spectrum for Precision (P) and Power (W) conditions at C3 electrode. Fig B. Correlation between Mu and Beta rhythms and MEPs. B1. Precision condition at t1; B2. Precision condition at t2; B3. Power condition at t1; B4. Power condition at t2. (PDF) [file pone.0207476.s001.pdf]
